# Paediatric acute rheumatic fever in developed countries: Neglected or negligible disease? Results from an observational study in Lombardy (Italy)

**DOI:** 10.3934/publichealth.2018.2.135

**Published:** 2018-05-23

**Authors:** Viorica Munteanu, Antonella Petaccia, Nicolae Contecaru, Emanuele Amodio, Carlo Virginio Agostoni

**Affiliations:** 1 Department of Paediatrics, IRCCS Ospedale Maggiore Policlinico, DISCCO, University of Milan, Milan, Italy; 2 Health Protection Agency of Brianza (Italy), Viale Elvezia n.2 Monza (MB) 20900

**Keywords:** acute rheumatic fever, epidemiology, hospitalization, children, adolescent, paediatric, Italy

## Abstract

**Introduction:**

Acute Rheumatic Fever (ARF) is a multisystemic disease that results from an autoimmune reaction due to group A streptococcal infection. The disease affects predominantly children aged 5 to 15 years and although its incidence in developed Countries declined since the early 1900s, to date there is a paucity of data that confirm this epidemiological trend.

**Objective:**

The study aimed to assess the burden of ARF in term of hospitalization and to describe the characteristics of acute rheumatic fever (ARF) in the paediatric population of Lombardy.

**Study design:**

The study was carried out by analyzing hospital discharge records of patients resident of Lombardy and aged 0–17 years old who, from 2014 to 2016, were hospitalized with the diagnosis of ARF. The following variables have been studied: age, sex, municipality of residence, date of diagnosis of each patient, hospital of admission, and presentation of the disease.

**Results:**

From 2014 to 2016, 215 patients were found to meet the inclusion criteria and diagnosed as affected from Acute Rheumatic Fever. The rate of hospitalization showed a slightly increasing trend from 3.42 in 2014 to about 5.0 in 2016. Moreover, ARF presented a typical seasonal trend with lower cases in the autumn and a peak of hospitalization in the spring.

**Conclusion:**

To date, ARF seems to be a rare but not negligible disease in southern central European countries, and in Lombardy we estimated an annual hospitalization rate of 4.24 cases per 100,000 children. The increasing trend found in our study suggests that the burden of the disease could be reduced by involving multidisciplinary health professionals who, in addition to the paediatrician of free choice, would promote evidence based medicine management of the disease during all its clinical phases.

## 1. Introduction

Acute Rheumatic Fever (ARF) is a multisystemic disease that results from an autoimmune reaction due to group A streptococcal infection. It occurs mainly in school-age children and young adults [1]. The most common and specific clinical manifestations of the disease affect joints, heart, brain, cutaneous and subcutaneous, and include polyarthritis, carditis, chorea, appearance of subcutaneous nodules and erythema or rash [2]. The most significant complication is cardiac involvement, also defined as Rheumatoid Cardiopathy (RC). The most important of its heart clinical manifestations are largely characterised by valvulitis, involving in particular the mitral valve and aortic valve, with chronic and disabling consequences.

Although the incidence and prevalence of ARF and RC are declining in developed Countries since the early 1900s, they continue to be the main causes of morbidity and mortality among young people in developing Countries. It has been estimated that there are over 15 million RC cases worldwide, with 282,000 new cases and 233,000 deaths each year [1]. Acute Rheumatic Fever affects predominantly children aged between 5 and 15 years of age. Initial episodes become less frequent in adolescents and young adults, and are rare in individuals over the age of 30; about 60% of people with ARF in endemic communities subsequently develop RC [3],[4]. The risk of ARF is fundamentally overlapping in males and females, but the risk of rheumatic carditis is 1.6 to 2.0 times higher in women [4],[5]. The latest estimates of the global number of rheumatoid cardiovascular patients include 9 million years of life lost adjusted for disability, 33 million prevalent cases and 275,000 deaths each year, which occur predominantly in low and middle-income countries [6]–[8]. RC prevalence increases with age [9], depending on adherence to secondary prophylaxis, severity of valve damage, access to specialist management and surgery [10],[11]. Acute Rheumatic Fever epidemiology varies from country to country [10], with a particularly high prevalence in Africa [12]–[14] and in the Pacific [15]–[18], in Latin America [19], the Middle East [20],[21] and in Asia [22],[23]. However, to date there is a paucity of updated data for several countries, including Italy. In this sense, updated data and both clinical and epidemiological characterization of the disease represent a major need for promoting preventive health strategies, quantifying national public health financial planning and, finally, reducing the burden of disease. According to the previous considerations, the main aim of this study was to examine the hospitalization rate attributable to ARF and describe its epidemiological characteristics in the paediatric population in Lombardy, the most populous region of Italy, during the 2014–2016 period.

## 2. Material and methods

Lombardy is one of the twenty administrative regions of Italy; it is sited in the northwest of the country, with an area of 23,844 square kilometres (9,206 sq. mi) and about 10 million people (population of 10,023,876 to 1 January 2017 from ISTAT data), representing one-sixth of Italy's population. Lombardy's capital is the second-largest city and the largest metropolitan area in Italy.

The study included a three-year timeframe, from January 1, 2014 to December 31, 2016. Subjects affected by ARF were identified by analysing hospital discharge records (HDRs) collected from all hospitals located in Italy and included in the Italian National Health Service. All data included in the analyses were obtained by the Italian Ministry of Health, which routinely collects HDRs from all the regions' hospitals. According to the national laws and considering that data were provided as anonymous records, individual consent was not required. Each HDR included demographic information (residence, gender, and date of birth), admission and discharge dates, discharge status (categorized as “discharged/transferred” or “expired”), and up to 6 discharge diagnoses (1 principal and 5 secondary diagnoses) coded according to International Classification of Disease, Ninth Revision, Clinical Modification (ICD-9 CM).

ARF cases were defined according to the following inclusion criteria: (a) occurrence of at least one ICD9-CM code of 390, 391.0, 391.1, 391.2, 391.8, 391.9, 392.0, 392.9, 393, 394.1, 395.0, 395.1, 395.2, 397.1, 397.9, 398.0, 398.90, 398.91, 398.99 either as principal or secondary diagnosis; (b) age < 18 years old; (c) resident of Lombardy. Hospitalization of Lombardy residents that occurred outside the region was also included in the analysis, while multiple hospitalizations due to transfers were combined. Admissions for transferred patients were followed until discharge. The following variables have been analyzed: age, sex, municipality of residence, date of diagnosis of each patient, hospital of admission, and presentation of the disease.

Statistical analyses were performed using the computing environment R (R Development Core Team, 2005) and maps were produced by ArcGIS (version 10.5.1 2016). Standard deviation was calculated for normally distributed quantitative variables. Hospitalization rates were calculated by dividing the number of hospitalizations by age cohort and then multiplying the quotient by 100,000. Two-sided P values and 95 percent confidence intervals were used to evaluate differences between males and females.

## 3. Results

**Figure 1.**
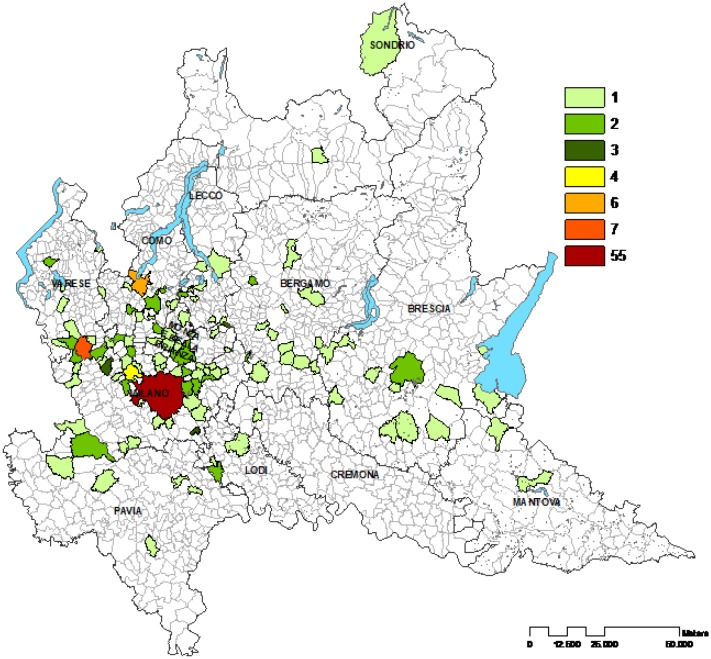
Geographic distribution of paediatric rheumatic cases in Lombardy (2014–2016).

From 2014 to 2016, 215 patients were found to meet the inclusion criteria and diagnosed as affected from Acute Rheumatic Fever. As reported in Table 1 and depicted in Figure 1, Milan was the first province for number of cases, accounting for 102 cases, followed by Varese: 22 cases, Como: 17 cases, Monza Brianza: 16 cases, Bergamo: 15 cases, Brescia: 13 cases, Lecco: 12 cases, Pavia: 9 cases, Lodi: 4 cases, Sondrio and Mantua (Mantova): 2 cases for province, and finally the Province of Cremona with only 1 case in three years.

**Figure 2.**
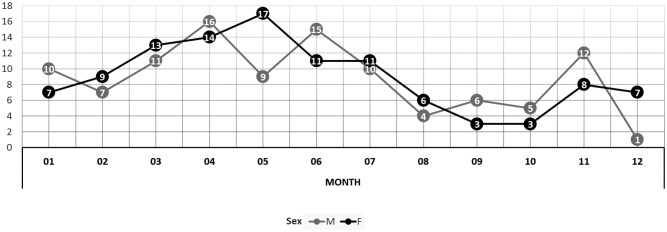
Number of hospital admissions divided by age and sex.

**Figure 3.**
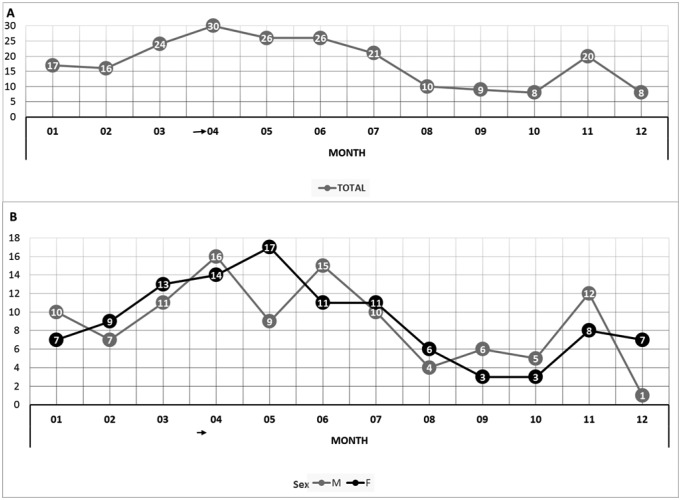
Number of hospital admissions divided by month of hospitalization in all patients (A) and in males vs females (B).

The hospitalization rate (cases/100,000 paediatric population) of ARF in Lombardy showed a slightly increasing trend from 2014 to 2016, from 3.42 cases in 2014 to about 5.0 cases in 2016. The mean age for patients was equal to 8.2 years (SD 3.1 years): 9.1 years in 2014, 10.3 years in 2015 and 7.4 years in 2016. As reported in Figure 2, 30 patients were younger than 5 years of age, accounting for 14% of the total (215 in Lombardy residents). Hospitalization rates were not statistically significantly different between males and females (4.45/100,000 *vs.* 4.30/100,000, respectively). Moreover, illness presented a typical seasonal trend with lower cases in autumn and then a steadily rises to its peak in April (Figure 3).

In 2014, the highest number of hospitalizations were diagnosed with “Chorea Rheumatic without mention of Cardiac Complications” (20% of total admissions) and “Chorea Rheumatic with Cardiac Complications” (18%) followed by “Acute Rheumatoid Endocarditis” (15%) and “Rheumatic Fever without mention of Cardiac Involvement” (10%).

In 2015, the diagnosis of “Acute Rheumatic Endocarditis” prevailed (17.7% of total admissions) followed by “Rheumatic Chorea without mention of Cardiac Complications” and “Rheumatic Fever without mention of Cardiac Involvement” (both 15.2%), and “Rheumatic Mitral Insufficiency” and “Unspecified acute Rheumatic Cardiac Disease” (12.7%).

In 2016, most patients were diagnosed with “Acute Rheumatic Endocarditis” (23.8% of total admissions), “Rheumatic Fever without Cardiac Complications” (16.7%) and “Chorea Rheumatic with Cardiac Complications” (14.3%).

**Table 1.** Summary of rates of incidence of paediatric Acute Rheumatic Fever per 100,000 residents.

Province	2014			2015			2016			2014–2016		
	Population	Cases	Rate * 100.000	Population	Cases	Rate * 100.000	Population	Cases	Rate * 100.000	Population	Cases	Rate * 100.000
*LECCO*	58.572	4	6,83	57.979	**5↑**	**8,62↑**	57.389	3	5,23	173.940	**12↑**	**6,9**↑
*MILANO*	529.174	25	4,72	527.147	**44↑**	**8,35↑**	529.011	**33↑**	**6,24↑**	1.585.332	**102↑**	**6,43↑**
*COMO*	101.075	1	0,99	100.825	3	2,98	100.227	**13↑**	**12,97↑**	302.127	**17↑**	**5,63↑**
*VARESE*	148.170	5	3,37	148.273	7	4,72	147.985	**10↑**	**6,76↑**	444.428	22	4,95
*PAVIA*	82.862	5	6,03	82.762	1	1,21	82.497	3	3,64	248.121	9	3,63
*MONZA BRIANZA*	148.994	9	6,04	148.884	2	1,34	148.826	5	3,36	446.704	16	3,58
*LODI*	39.094	0	0	39.021	1	2,56	38.867	3	7,72	116.982	4	3,42
*BERGAMO*	203.630	4	1,96	202.890	**2↓**	**0,99↓**	201.212	9	4,47	607.732	15	2,47
*SONDRIO*	30.247	0	0	29.880	1	3,35	29.561	1	3,38	89.688	2	2,23
*BRESCIA*	228.002	3	1,32	226.986	7	3,08	225.196	**3↓**	**1,33↓**	680.184	**13↓**	**1,91↓**
*MANTUA*	68.171	1	1,47	67.988	**0↓**	**0↓**	67.433	1	1,48	203.592	**2↓**	**0,98↓**
*CREMONA*	57.783	1	1,73	57.492	**0↓**	**0↓**	57.115	**0↓**	**0↓**	172.390	**1↓**	**0,58↓**

***LOMBARDIA***	1.695.774	**58**	**3,42**	1.690.127	**73**	**4,32**	1.685.319	**84**	**4,98**	5.071.220	**215**	**4,24**

↑: high incidence rate by column, ↓: low incidence rate by column.

## 4. Discussion

Acute Rheumatic Fever (ARF) confirms to be still a current public health problem in Italy and to date it can be not considered a negligible disease in developed Countries. The real doubt is, therefore, if it can be considered a neglected disease. The question is difficult to answer but this study offers some findings that could help to clarify epidemiology of ARF among Italian paediatric population requiring hospitalization. A first finding of interest is that a significant number of paediatric ARF cases were recorded during each of all three years of the study, showing also an increasing trend from 2014 to 2016. We are well aware that three years are probably too short a period for evaluating trend but, noteworthy, there was no year without cases, suggesting that in Lombardy ARF shows to have the characteristics of an “endemic” disease with low incidence. Moreover, if the observed trend should be confirmed in future years, it would represent an inversion with respect to the past century when a decline in the incidence of acute rheumatic fever in developed countries has been observed as consequence of improved public hygiene and widespread use of antibiotics. To date, ARF seems to be a rare but not negligible disease not only in Lombardy, where we estimated about 4 cases per 100,000 children requiring hospitalization because of ARF or ARF complications, but also in other European countries. In this sense, our estimates are quite similar to those observed in Abruzzo, another Italian region, in the period 2000–2009, where 88 cases were found with an average annual incidence rate of 4.10 cases for 100,000 years-child [5]. Otherwise, the recent ARF outbreak in Slovenia revealed that the disease is still present in southern central Europe with an estimated annual incidence of 1.25 cases per 100,000 children [24]. These last data seem to be lower than those reported in our experience and in the other study previously cited and carried out in Abruzzo. The reasons for this difference could be attributed, at least in part, to the different study methods and we cannot exclude that some ARF cases reported in our study are not incident cases but prevalent ones. However, this hypothesis would explain only a limited part of the difference and further investigations may be required for clarifying these regional variations.

Differently, no significant regional variations with respect to other studies were found in distribution of ARF complications. Our data showed that rheumatic carditis was the most frequent complication accounting for 61% of all diagnosis, which is similar to the other published data, in which it ranges from 45% to 95% [25],[26]. Also the increase in the rate of hospitalization of patients with ARF, from March to July, with a peak in April and a second mild peak in November has been reported by other authors and it is in agreement with the seasonal variability of pharyngitis, which is caused by group A beta-haemolytic streptococci and is most often diagnosed in the winter and spring [27] or in the fall and winter [28],[29]. Sydenham's chorea was reported in 26% of cases, although some authors observed rates to a minimum of 15–17% [30]. Some publications indicate rates of 6–31% and even 49% [31].

Finally, the mean age of patients with ARF in our study corresponds to the age reported by other authors [32]. Our study could be affected by some limitations that should be taken into account as the possibility that some ARF cases have not been hospitalized and thus not included in our analysis. Moreover, the lack of information about patient nationality could have underestimated the contribution of immigration in a region as Lombardy that, in the past decades, has received a very high number of people especially from Africa [12]–[14]. Unfortunately, the precise picture of the disease burden attributable to this microorganism is difficult to quantify, especially in contexts such as the Italian one, where ARF is not included among the diseases subject to notification. In such a context, hospital discharge databases may be the only way to understand, accurately and economically, the epidemiology of this disease. Despite these possible limitations, at our best knowledge this is the largest population study carried out in an Italian region. Two main messages come from our experience. The first is that ARF is a disease that cannot be considered eliminated in Lombardy and the second one is that, surprisingly, an increasing trend should stimulate further studies on this topic.

## 5. Conclusion

AFR is one of the main causes of heart disease among young people and it is still present in developing countries, but not only in these. In recent decades, there has been a new increase in ARF incidence in European countries and results obtained in the present study unquestionably agree with this datum. The increasing trend that we have found also suggests the needs for multidisciplinary, specialist, paediatric approach involving several professionals who, in addition to the paediatrician of free choice, promote evidence based medicine management of the disease during all phases of ARF. The involvement of the paediatrician is fundamental, both to prevent the disease and to follow the young patients affected by this disease. Every paediatrician should always bear in mind the risk of ARF and, therefore, should not underestimate even first symptoms attributable to a possible infection with group A beta-hemolytic Streptococcus.

However, ARF management, which involves patients since their childhood, requires coordination, often throughout life, between prevention, diagnosis, treatment, psychological, rehabilitative and social assistance. For these reasons, it will be essential to develop appropriate tools for organization and communication in this area.
